# An unsupervised neuromorphic clustering algorithm

**DOI:** 10.1007/s00422-019-00797-7

**Published:** 2019-04-03

**Authors:** Alan Diamond, Michael Schmuker, Thomas Nowotny

**Affiliations:** 1 School of Engineering and Informatics, University of Sussex, Falmer, Brighton, BN1 9QJ UK; 2 Department of Computer Science, University of Hertfordshire Hatfield, Hertfordshire, AL10 9AB UK

**Keywords:** Neuromorphic hardware, Self-organizing map, Data clustering, Unsupervised learning, Spiking neural networks, Classification

## Abstract

Brains perform complex tasks using a fraction of the power that would be required to do the same on a conventional computer. New neuromorphic hardware systems are now becoming widely available that are intended to emulate the more power efficient, highly parallel operation of brains. However, to use these systems in applications, we need “neuromorphic algorithms” that can run on them. Here we develop a spiking neural network model for neuromorphic hardware that uses spike timing-dependent plasticity and lateral inhibition to perform unsupervised clustering. With this model, time-invariant, rate-coded datasets can be mapped into a feature space with a specified resolution, i.e., number of clusters, using exclusively neuromorphic hardware. We developed and tested implementations on the SpiNNaker neuromorphic system and on GPUs using the GeNN framework. We show that our neuromorphic clustering algorithm achieves results comparable to those of conventional clustering algorithms such as self-organizing maps, neural gas or *k*-means clustering. We then combine it with a previously reported supervised neuromorphic classifier network to demonstrate its practical use as a neuromorphic preprocessing module.

**Electronic supplementary material:**

The online version of this article (10.1007/s00422-019-00797-7) contains supplementary material, which is available to authorized users.

## Introduction

Spiking neural networks in brains can solve many challenging problems while consuming surprisingly little energy. Their biological components run relatively slowly but rely upon very high parallelization alongside powerful information coding and network topologies, which have emerged through evolution. Highly parallel and power-efficient “neuromorphic platforms,” which mimic these biological mechanisms, have been developed as a basis for brain-like computing in the domain of machine learning and artificial intelligence (AI), for example TrueNorth (Merolla et al. 2014), SpiNNaker (Khan et al. 2008; Furber et al. 2013), Neurogrid (Benjamin et al. 2014), Minitaur (Neil and Liu 2014), Loihi (Davies et al. 2018), DYNAPs (Moradi et al. 2018) and the “BrainScaleS” system (Schemmel et al. 2010). These systems support the simulation of up to millions of modeled spiking neurons and billions of synapses in real time, i.e., at the same speed that neurons operate in the brain. The BrainScaleS platform even operates $10^4$ times faster than real time and thus supports accelerated network simulations.

“Neuromorphic algorithms” are being developed that leverage these platforms to solve new and old computing problems from the fields of AI and data mining. One class of such problems is clustering. To date, there are no definitive solutions for neuromorphic hardware that could compare to classical algorithms such as self-organizing maps, neural gas or *k*-means clustering (Xu and Wunsch 2005). This may partially be due to the constraints on network connectivity and, more importantly, learning rules, which currently available hardware imposes. The most commonly supported learning rule, for instance, is spike timing-dependent plasticity (STDP), which updates synaptic weights based on the temporal relationship of pre- and post-synaptic spikes (Gerstner et al. 1996; Markram et al. 1997; Bi and Poo 1998, 2001). The input to the learning rule is hence strictly local to the synapse. Spiking self-organizing maps have been proposed (Choe and Miikkulainen 1998; Ruf and Schmitt 1998; Behi and Arous 2011), but their weight update rules depend on non-local information and are hence not compatible with STDP rules that are available on current neuromorphic platforms. There is, however, a body of previous work (Nowotny et al. 2005; Masquelier et al. 2009; Nessler et al. 2013), carried out using conventional simulations of neurons and relying only on local STDP, that suggest potential neuromorphic-compatible solutions.

In this study, we demonstrate a neuromorphic implementation of a self-organizing network that learns typical activation patterns (*“prototypes”*) in a multivariate dataset, relying only on local STDP. We show that correlations between similar inputs are sufficient to drive fully unsupervised learning that leads to individual output neurons to “represent” particular prototypes. Lateral inhibition plays a critical role in the self-organized learning process as it supports a (potentially multiple) *winner-take-all* (WTA) condition between neurons. Together with the employed STDP rule, this causes input synapses of the neurons that respond strongest to a given input pattern to potentiate, effectively reinforcing the neurons’ affinity to the input pattern. Likewise, neurons that respond weaker are inhibited from firing and, consequently, reduce their affinity to the pattern. The result is a set of disjoint subpopulations within the population of output neurons, which approximately represent, through their input weights, centroids of inherent clusters in the space of inputs. We demonstrate the operation of the self-organizing network on the GeNN GPU-accelerated simulator and the SpiNNaker neuromorphic hardware system, learning prototpyes of handwritten digits in the MNIST dataset (http://yann.lecun.com/exdb/mnist/).

## Methods

Neuromorphic algorithms are essentially computational models of neuronal networks, and we here use standard computational neuroscience models to build neuromorphic algorithms for clustering.

*Neuron and synapse models* Our model for unsupervised clustering and subsequent classification was implemented in both the GPU-enhanced neuronal network (GeNN) framework (Yavuz et al. 2016; Nowotny et al. 2014) and the SpiNNaker platform (Khan et al. 2008; Furber et al. 2013). We used different neuron and synapse models for the GeNN and SpiNNaker platforms. On one hand, this was done because of restrictions in the two systems that constrain the preferred choice of model. On the other hand, we think that replicating the same behavior with different models emphasizes the robustness and reproducibility of the proposed functional network model. Neuron and synapse models are detailed in Diamond et al. (2016a, b).

In brief, inputs to the network were described by Poisson processes with rates $\lambda _i$ that depend on the identity of the input *i*. The relationship between the rates and the input identity is described in more detail in the “Network model” section. The Poisson processes were approximated as Bernoulli processes with $p_i = \lambda _i \cdot \varDelta t$ for fixed time steps $\varDelta t$, where $\varDelta t = 1\;$ms for SpiNNaker and $\varDelta t= 0.5\;$ ms for GeNN.

For all other elements, we used the most lightweight neuron and synapse models available on each of the two platforms. For GeNN, all neurons that are not Poisson processes were modeled as “map” neurons (Rulkov 2002),

1 $$\begin{aligned} V(t+\varDelta t)= \left\{ \begin{array}{ll} \frac{V_{{\text {spike}}}^{2}\alpha }{V_{{\text {spike}}}(1+y)-V(t)-\beta I_{{\text {syn}}} (1-m)} &{}\quad \text {if } \; V(t) \le 0\;\text {mV} \\ V= V_{{\text {spike}}}(\alpha +y) &{}\quad \text {if } 0 < V(t) \le V_{{\text {spike}}}(\alpha +y), \\ V(t-\varDelta t) \le 0 \\ - V_{{\text {spike}}} &{}\quad \text {otherwise}\nonumber \\ \end{array}\right. \\ \end{aligned}$$

2 $$\begin{aligned}&m(t+\varDelta t)= (1 - a \varDelta t) m(t) \end{aligned}$$

3 $$\begin{aligned}&\text {A spike is emitted when } V= V_{\text {spike}}(\alpha +y) \end{aligned}$$

4 $$\begin{aligned}&\text {On spike, } m \mapsto m + b(1-m), \end{aligned}$$

where $$V_{\text {spike}} = 60\;$$mV scales the spike amplitude ($$=V_{\text {spike}}\cdot (1+\alpha +y)=91.92\;$$mV top to bottom), $$\beta = 0.0165\;$$mV/nA reflects the input resistance of the neurons, $$y= -2.468$$ regulates the intrinsic excitability of neurons and $$\alpha = 3$$ regulates the spike shape. *m* implements a form of spike rate adaptation where the neurons become less sensitive to inputs if they spike frequently. $$a= 10^{-4}\;($$ms$$)^{-1}$$, $$b= 0.02$$ and $$\varDelta t =0.5\;$$ms.

Synapses were all modeled as conductance-based synapses,

5 $$\begin{aligned} I_{\text {syn}} = g S (V-V_{\text {syn}}) \end{aligned}$$

where *g* is the maximal synaptic conductance of each synapse, *S* is its activation and $V_{\text {syn}}$ is the synaptic potential, $V_{\text {syn}} = 0\;$mV for excitatory and $V_{\text {syn}} = -\,92\;$mV for inhibitory synapses. Activation is governed by

6 $$\begin{aligned} S(t+\varDelta t)= \left\{ \begin{array}{ll} S(t) + 1 &{}\quad \text {if presynaptic spike} \\ \left( 1 - \frac{\varDelta t}{\tau _{\text {syn}}}\right) S(t) &{}\quad \text {otherwise} \end{array} \right. \end{aligned}$$

The entire network model was simulated with a global timestep of $0.5\;$ms, as dictated by the Rulkov map neurons (Rulkov 2002).

On SpiNNaker, neurons were modeled as leaky integrate-and-fire (LIF) neurons (Rast et al. 2010).

7 $$\begin{aligned} \frac{\text {d}V}{\text {d}t} = \frac{1}{\tau _m} (V_{\text {rest}} - V + I_{\text {syn}} R) \end{aligned}$$

with membrane time constant $\tau _m= 20\;$ ms and $V_{\text {rest}} = -66\;$mV. If $V \ge -\,65\;$mV, a spike is emitted and *V* is reset to $V_{\text {reset}} = -\,70\;$mV. The neuron is then kept at $V_{\text {reset}}$ for a refractory period of $2\;$ms.

Synapses were described by exponential current-based synapse models, i.e.,

8 $$\begin{aligned} \frac{\text {d}I}{\text {d}t} = -\frac{I}{\tau _{\text {syn}}} + g_{\text {syn}} \delta (t_{\text {spike}}) \end{aligned}$$

where $t_{\text {spike}}$ denotes the time of a presynaptic spike and $\delta$ is the $\delta$ distribution. $g_{\text {syn}}$ is measured in *nA* and represents the synaptic strength. The equations for neurons and synapses are integrated in a variant of an Euler algorithm within SpiNNaker’s bespoke 32-bit/16-bit fixed point arithmetic (Rast et al. 2010). On SpiNNaker, the model was simulated with a $1\;$ms integration time step.

*Spike timing-dependent plasticity* STDP was implemented in excitatory synapses for both the unsupervised learning between input and the first neuron layer and the reinforced learning from this layer to association neurons, which represent the output of the network (see below). The change $\varDelta g$ applied to the conductance *g* of a synapse between a pre- and a post-synaptic neuron depends on the interval between their firing $\varDelta T= t_{\text {post}}-t_{\text {pre}}$,

9 $$\begin{aligned} \varDelta g = \left\{ \begin{array}{ll} -\,0.0125\;\upmu \text {S} &{} \quad \text {if } 20\;\text {ms}< \varDelta T \le 200\;\text {ms} \\ -\,0.0117 \frac{\upmu \text {S}}{\text {ms}}\varDelta T + 0.223\;\upmu \text {S} &{} \quad \text {if } 2\;\text {ms}< \varDelta T \le 20\;\text {ms} \nonumber \\ -\,0.0025 \;\upmu \text {S} &{} \quad \text {if } -200\;\text {ms} < \varDelta T \le 2\;\text {ms} \\ 0\;\upmu \text {S} &{} \quad \text {otherwise} \end{array} \right. \\ \end{aligned}$$

**Fig. 1:**
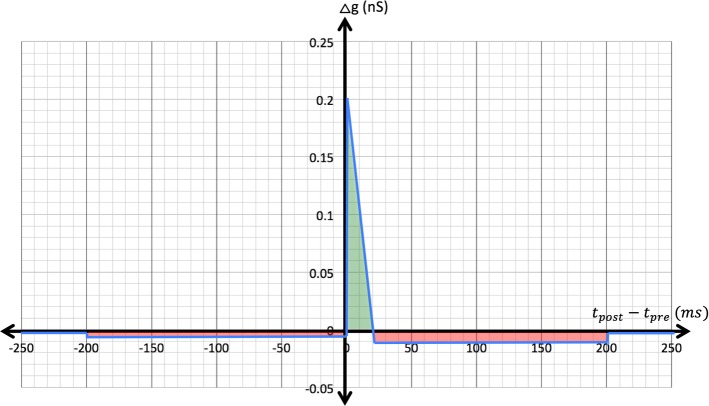
Configuration of spike timing-dependent plasticity (STDP). The blue trace shows conductance changes—i.e., synapse potentiation (green zone) or depression (red)—applied across the pertinent range of spike intervals between firing of a presynaptic neuron and a downstream, connected post-synaptic neuron

The relationship of $\varDelta g$ to the firing interval $\varDelta T$ is illustrated in Fig. 1, and the motivation for its shape is detailed in the model explanation below.

Synapse conductance was clipped at an upper limit $g_{\text {max}}= 0.25\;\upmu$S and at a lower limit of $g_{\text {min}}=0\;\upmu$S.

## Network model

**Fig. 2:**
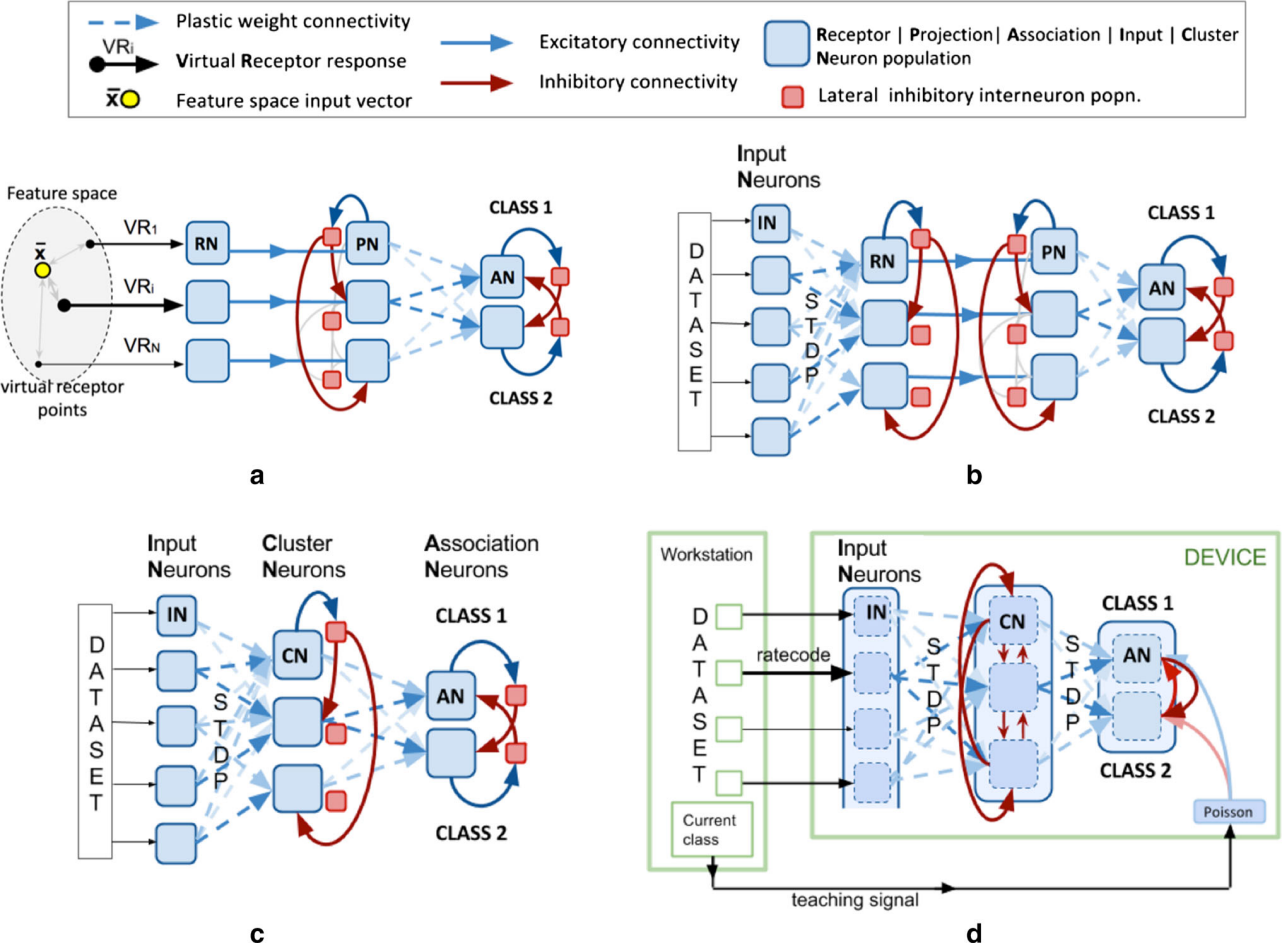
Neuromorphic self-organized mapping and spiking classifier model. **a** Previous conceptual neuromorphic algorithm for a generic multivariate classifier design based on the insect olfactory system. Inputs were clustered using a (unsupervised) neural gas algorithm on a standard PC up front leading to VR$_1$ to VR$_N$. **b** Adapted conceptual model using the neuromorphic self-organized mapping (unsupervised learning stage). The previously used clustering of the input space to produce a set of virtual receptor points is replaced with the self-organizing set of RN neurons that are tuned to represent prototypes in the input space during a first pass through the training set. **c** The new model optimized for size whereby the RN and PN layers are merged into a single self inhibiting layer, now labeled CN (cluster neurons). **d** The final implemented model optimized further: Layers are now implemented as single populations with subpopulations demarcated by connectivity and the biologically correct inhibitory interneuron populations are made redundant by using direct inhibitory synapses between subpopulations. An additional population of Poisson neurons was added to implement supervised learning during the second phase of training, by exciting or inhibiting designated output subpopulations depending on the class of the presented input (“teaching signal”). The number of output neurons reflects the number of classes, e.g., 10 for the MNIST dataset; only 2 are shown in the figure for simplicity

Self-organized clustering is achieved with a simple two-layer network of input and output neurons, connected by STDP synapses (layers “IN” and “RN” in Fig. 2b, and “IN” and “CN” in Fig. 2c, d). All functional units of the network are small populations of individual model neurons that we refer to as groups. In order to test the performance of the neuromorphic self-organized mapping produced by our model, we also created a classifier stage adapted from an existing design that had employed offline machine learning to perform equivalent data clustering (Schmuker and Schneider 2007; Diamond et al. 2016a, b; Schmuker et al. 2014).

Figure 2a illustrates the original model (Schmuker et al. 2014). The first layer comprises groups of receptor neurons (RNs) encoding multivariate, real-valued data samples $\mathbf s \in {\mathbb R}^d$ into a population-based, positive, bounded, firing-rate representation ${\varvec{{\lambda }}} \in {\mathbb R}_+^{N_{RN}}$. Each RN was modeled as a Poisson process with firing rate

10 $$\begin{aligned} \lambda _i= \lambda _{\text {max}} \left( 1-\frac{\text {d}(\mathbf s ,\mathbf p _i)-\text {d}_{\text {min}}}{d_{\text {max}}-d_{\text {min}}}\right) \end{aligned}$$

where $\text {d}(\mathbf s ,\mathbf p _i)= \sum _{j=1}^d |s_j - p_{i,j}|$ is the Manhattan distance (L1 metric) between an input $\mathbf s$ and the coordinates $\mathbf p _i$ of the *i*th “virtual receptor” (VR). This terminology was motivated by the similar functional role of receptors in the olfactory system. $d_{\text {min}}$ and $d_{\text {max}}$ are the minimal and maximal distances observed in the dataset from any VR, so that $\lambda _i$ ranges from 0 to $\lambda _{\text {max}}= 40\;$Hz. The coordinate vectors $\mathbf p _i$ of the VRs were determined up front using a neural gas clustering algorithm (Martinetz and Schulten 1991) to partition the input space to a specified resolution. Effectively, this approach encodes the data using cone-shaped radial basis functions with large, overlapping receptive fields. Each group of RNs excites a matching group of “projection neurons” (PNs) in the second layer, which inhibits all other PN groups by lateral inhibition. This reduces correlation between VR channels and sparsens the representation of the multi-dimensional pattern.

The third layer of “association neuron” (AN) groups acts as a readout for the classifier, whereby each AN population is assigned to signal the presence of a given class in the input. Patterns of PN activity are trained to correlate with firing of the correct output association neuron (AN) group by linking them with plastic connections and activating the correct output group during training. Connection strengths $g_{\text {PN}\,\text {AN}}$ are altered using spike timing-dependent plasticity (STDP) (see above).

Figure 2b illustrates the fully neuromorphic model that is the subject of this paper. The VR proximity function is now replaced with an initial processing stage that is neuromorphic. The model receives the raw input data $\mathbf s$ in their full dimensionality *d*. The entries of $s_j \in [0,1]$ of $\mathbf s$ are the gray levels of pixels of the 28x28 MNIST images, and each group of input “neurons” IN (Fig. 2b) contains Poisson processes with rate $\lambda _j = \lambda _{\text {max}}s_j$. STDP in IN to RN connections and lateral inhibition between RN groups leads to RN, and hence PN groups to respond to a representative range of input patterns, after one or more initial passes over the training set.

The STDP curve (Fig. 1) was constructed such that each group of RN neurons eventually responds selectively to a common representation or rendering of one of the classes (digits 0–9 in this case) in the training dataset. The IN-RN weights are initialized with a random, but high, value. During self-organized clustering, synapses from active INs (dark pixels) are potentiated, but more importantly, synapses from less active INs (white pixels) are strongly depressed. As a result, the weights migrate toward a “mirror-image” in weight space of the class prototype that is represented by the RN group in question.

For the learning curve, this implies that, as in earlier work (Nessler et al. 2013), synapses are only potentiated in a short window ($\sim 20$ ms) when post-synaptic firing closely follows presynaptic firing. As with classic STDP, the slope recognizes the fact that causality becomes less likely as the interval increases. If post-synaptic firing occurs later, it is treated as non-causal and synapses are depressed by a fixed amount ($\varDelta g= -\,0.0125\,\upmu$S). If post-synaptic firing precedes presynaptic firing, then it is also considered non-causal and synapses are always depressed by a small amount ($\varDelta g= -\,0.0025\,\upmu$S). In the classic STDP rule (Bi and Poo 1998), strong depression is applied for very close post-then-pre spiking. However, when using populations of noise-driven neurons, many coincidental, close spike pairings between disparate neurons are generated and the order of pre- and post-synaptic spiking is quite variable. Over time, a symmetrical depression window for post-then-pre spike pairings as in the original STDP rule results in a net depression of all synapses. In effect, all weights fade away. For similar reasons, we did not apply depression for large $\varDelta T$ exceeding 200 ms in duration. Without this limit, irrelevant spike pairings across different input presentations tend to cause long-term depression of all synapses and hence learned connections gradually fade away.

The result of this strategy is that input patterns will be stored in the input weight space of a responding neuron group. The initial high weighting, the lateral inhibition between groups and the length of presentation can all be configured and tuned to minimize overlearning—“burning in”—of a single input pattern and to allow activity to switch to alternative groups for novel input patterns.

As a result of learning at this initial stage, the input is mapped into $N_{\text {RN}}= N_{\text {PN}}$ dimensions, where $N_{\text {RN}}$ and $N_{\text {PN}}$ are the number of RN and PN groups, respectively.

It is evident from Fig. 2b that the RN and PN layers are implementing a very similar function with the primary difference that the strength of lateral inhibition is set for two different purposes. For the RN layer, it is set to high values in order to implement effective WTA during the self-organized mapping.

For the PN layer, once the representation in RNs is established, inhibition is then used to only mildly reduce correlations between clusters and sparsen the representation of the multi-dimensional patterns. A strict WTA behavior is no longer necessary or, indeed, desirable. Figure 2c shows how we can significantly reduce the model size (neuron and synapse count) by combining RN and PN layers into one, which we label simply “cluster neurons” (CNs). After unsupervised training of the input mapping via the input weights, the role of CNs can be switched by decreasing the inhibitory weights before embarking on the second supervised stage of training the output layer.

Figure 2d shows the implemented final model, which incorporates optimizations to further reduce the model size, and the Poisson population that enables supervised training of the readout association layer (AN) (see below).

*Connectivity and population sizes* As already mentioned, the model was implemented in both the GPU-enhanced neuronal network (GeNN) framework (Yavuz et al. 2016; Nowotny et al. 2014) and the SpiNNaker platform (Khan et al. 2008; Furber et al. 2013). For efficient definition on both platforms, each layer was implemented as a single population containing neuron groups demarcated solely by their connectivity. Following the unsupervised training of the map, the subsequent supervised association training of the output layer is undertaken in order to encourage firing in the correct output. To induce appropriate associative potentiation of synapses when an input of a given class is presented, a further 40 neuron Poisson input population with a constant $\lambda _{\text {teach}}= 60$ Hz firing rate is employed, effectively generating a teaching signal consisting of excitation and inhibition onto the ANs. By injecting additional excitatory synaptic current from the Poisson population, we increase the likelihood that the correct AN group will spike in response to the current CN activity. The resulting correlated firing of CN and AN neurons causes STDP-based weight potentiation in the corresponding CN-AN synapses. Conversely, sending the spikes of the teaching population via inhibitory synapses to incorrect AN groups discourages output firing, preventing incorrect associations. The teaching signals are removed during testing.

Input (IN) groups contain 10 neurons each, and output (AN) groups contain 40 neurons each to ensure effective WTA (Diamond et al. 2016a, b). CN group sizes varied and were investigated between 10 and 60 neurons.

Connectivity between subpopulations, either within or across layers, is universally random with probability of connection $p_\mathrm{c}= 0.5$, except for the IN-CN input connections which have probability $p_c^\mathrm{IN\,CN}= 0.75$.

*Initial weights* STDP synapses are initialized to a high conductance value (i.e., strongly weighted) by selecting weights from a uniform distribution between $\frac{g_{\text {max}}}{2}$ and $g_{\text {max}}$. In conjunction with the STDP rule described above, this choice ensures that when a CN neuron group begins to represent a prototype input pattern, its average synaptic strength remains comparable to, if not lower than, that of naive CN groups, and hence, dissimilar input patterns will be represented by other CN groups. Inhibitory WTA synapses are fixed at $0.025\;\upmu$S, while the CN layer is first exposed to inputs to ensure that typically only one CN neuron group will respond to each input pattern at this stage. When the CN layer has developed a representation of the input space, the CN-CN synapses are reduced to $0.015\;\upmu$S to produce more gentle lateral inhibition and a multiple WTA representation.

*Training and test regimes* Inputs were presented by setting the appropriate firing rates in the INs. A “silence” gap of $50\;$ms (simulated) was introduced between each presentation of an input pattern to ensure that inter-spike intervals triggering weight plasticity do not occur between spikes relating to two different input patterns.

During training, each input is presented until the number of spikes generated in the CN layer reaches a specified limit. This setting can be varied usefully between 20 and 200 spikes and has a significant effect on the performance of both clustering (mapping) and eventual classification (see Sect. 4). We introduced this mechanism because if a well-established input pattern is presented, this causes an immediate spiking response in one or more well matched AN groups. Using the spike limit allows to reduce the amount of additional synaptic plasticity that occurs before switching to the next input. Conversely, a novel input will initially trigger only low, or no, spiking and it is useful to leave the input pattern in place until the response has built up to a more commensurate degree. As a result, the system skips rapidly over known inputs applying only minor modifications to synapses that unknown stimuli are presented longer, which favors learning novel inputs. The net effect in terms of mapping the input space is to pull the emerging “map” of clusters toward less charted areas of feature space.

During testing, the spike-limited switching is replaced by a time-based regime where each input is presented for exactly $50\;$ms. This allows all output subpopulations the same time to generate sufficient spikes to allow consistent classification. Retaining the low CN spike count limits used in the training stage as a driving mechanism was found to lead to insufficient output activity for reliable classification or WTA action between AN subpopulations.

The classification decision of the model is calculated by determining the AN group with the most spikes in the time window from the input presentation until the end of the subsequent 50ms silent period imposed on the INs. This is important as there is a delay between spikes appearing at the IN layer and the spike response at the final AN output layer.

The training set is presented to the network exactly two times. The first epoch is used to allow self-organization of the CN layer (IN to CN weightings). For the second epoch, the lateral inhibition is reduced to $0.015\;\upmu$S, as described in Methods, and the association layer (CN to AN weightings) is also trained. During this stage, a teaching signal is introduced from the teacher population that activates the correct output neurons for each input. While learning in the brain is more likely to be gated by neuromodulatory signals acting on the synapses (Urbanczik and Senn 2009), activating an appropriate output neuron in conjunction with a Hebbian learning mechanisms to implement supervised learning is a well-known technique in artificial neural networks. On GeNN, the routing of the teaching signal to the output AN populations is implemented by switching the appropriate excitatory and inhibitory synapse conductances to either $0\;\upmu$S or $0.5\;\upmu$S. On SpiNNaker, changing weights mid-simulation is not supported. Instead, separate 10 neuron input populations (one per class) are used to pass in the teaching signal (see Diamond et al. 2016a for details). During the test stage, all teaching signals are disabled.

Note that all plasticity mechanisms remain in place throughout training and testing. This is partly imposed by inflexibility in both platforms which do not allow for structural model changes during a contiguous run. However, it is also not necessarily unrealistic compared to biological learning, where there is no clear distinction between training and testing. The only exceptions to continuous learning in our model are that output CN-AN weights are reset following the first training set exposure and that the lateral inhibition between CNs is reduced during the second presentation of the training set. The supervised learning in CN-AN connections hence is only relevant during the second exposure, and the competition between CN neurons is less pronounced in this phase. Initial investigations showed that markedly improved results are obtained if self-organization in the CN layer reaches a certain level of completion first before ANs begin to develop their receptive fields.

*Implementation* For GeNN, the compiled CUDA-based model and initial data were uploaded to the GPU and stepped at maximum speed under control of the host workstation. The desired spiking rates for Poisson populations were uploaded as a block and revised at will. For GeNN, we used a workstation (8-core, 3.7 Ghz Intel Pentium Xeon, 32 GB RAM) installed with an NVIDIA Titan Black GPU card (2880 cores, 6 GB memory). This is classified as a high end consumer/gaming product, connected internally via PCI-Express bus. Note that a second small video card was used to drive the workstation’s main display, freeing up the main GPU. We used NVIDIA CUDA 7.0 and the GeNN 2.1 software release supplied by University of Sussex (Nowotny et al. 2014; Yavuz et al. 2016). Simulation speed ranged from 5 to 20 times real time, depending on the model size. Spike count data of interest were downloaded from the GPU device between steps.

For SpiNNaker, the model was defined using the PyNN modeling toolkit (Davison 2008) on the host workstation. When the simulation was invoked, the model was compiled, uploaded and set to run in real time (1 simulated second = 1 elapsed second), managed by the sPyNNaker toolset (Rowley et al. 2015). As there is no stepping or pausing, the training and test stages were performed contiguously in a single run; multi-channel (28$\times$28 dimensions) input spikes were passed in real time from the host machine using the live spiking UDP package interface. Spikes produced by the CN layer neurons (needed for spike count limiting) and the AN layer neurons (needed for the classifier’s verdict in testing) were collected live on the host using the UDP output spiking interface. Note that the real-time multi-channel input and output is handled by host-based software we developed for SpiNNaker simulations with datasets larger than can be usefully handled by the spike source file interface. This software is available on GitHub (Diamond 2016).

We used a “SpiNN-5” board hosting 48 SpiNNaker chips with 18 ARM9 cores each. The board was connected directly to the same workstation as was used for the GPU simulations via 100 Mbps Ethernet. The SpiNNaker board was provided by Steve Furber’s group, University of Manchester, UK. We used the sPyNNaker software base supplied by Manchester (Rowley et al. 2015), release “Little_Rascal.”

*Test dataset for validation* To test the model, we used the benchmark MNIST digit classification task (http://yann.lecun.com/exdb/mnist/). This was chosen, firstly, as a standard non-trivial classification problem, which must handle both a relatively high number of samples (tens of thousands) and also high dimensionality (images are grayscale, 28 $\times$ 28 pixels = 784 dimensions) as far as this model is concerned (see earlier discussion around pixel independence). Secondly, by choosing an image-based task, we are able to meaningfully visualize and thus report the details of the incremental learning taking place within the weight space of the model’s synapses.

By default, MNIST digits are encoded as horizontal rows of pixels, moving from top left to bottom right. These are mapped as neurons 0 to (10 $\times$ 784) in the input layer, using groups of 10 neurons per pixel as described above.

Initial investigations showed that a larger number of CN groups necessitates more input data in order to train them usefully. For the results shown here, we used $N_{\text {train}}= 0.4\,\mathrm{K}$ training digits per class for *K* CN groups, motivated by an initial parameter sweep. For mapping with 100 CN groups, this implies a training set of 40 digits per class. For each of 5 runs, test digits were presented stratified (0,1,2$\ldots$). In order to avoid over-fitting (Nowotny 2014), all initial investigations and parameter adjustments were performed using cross-validation on a randomly chosen subset of the MNIST training set and the final results reported here are based on a subset of the entirely separate MNIST test set. In this case, 10,000 digits (training 1600 $\times$ 5, test 400 $\times$ 5) were drawn randomly from the corresponding MNIST datasets.

## Results

We tested our model by comparing its clustering performance against conventional machine learning before seeking to demonstrate its practical use as a neuromorphic data processing module with our published generic neuromorphic linear classifier (Schmuker and Schneider 2007; Schmuker et al. 2014; Diamond et al. 2016a, b).

**Fig. 3:**
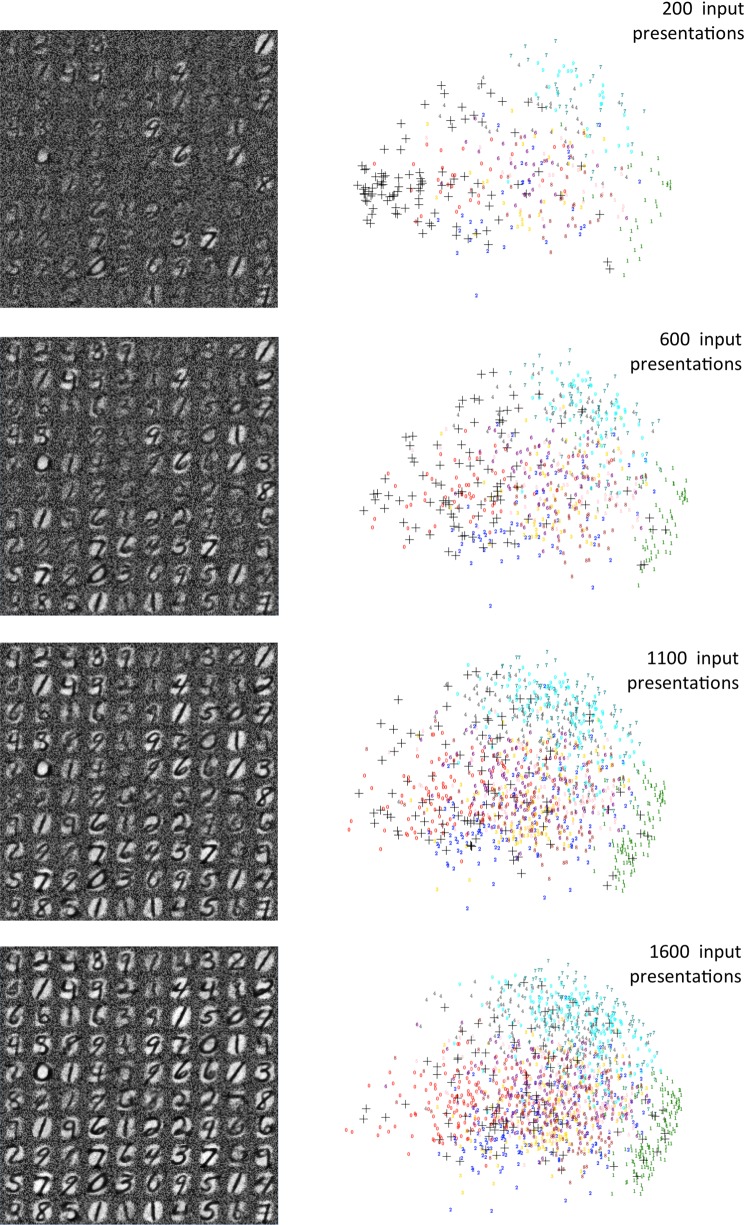
Visualization of stages in self-organized mapping out of 100 cluster points in MNIST feature space over the course of presentation of 1600 training digits. The left column shows, at 4 stages, the average weight of plastic synapses from each rate-coded input pixel to each of the 100 neuron subpopulations. The right column shows the learned cluster points (black crosses) in a 2D PCA projection of feature space overlaying the set of data points presented up to that stage (colored digits). As inactive pixels in the input will retain their high initial weighting, we first masked out (neglected) the least active 10% of pixels across the training set in order to meaningfully plot the clusters’ points against the input dataset

### Clustering performance

Figure 3 shows the development of 100 clustering points across the course of a training run consisting of two presentations of 1600 training digits. To obtain a visual “pseudo-digit” representation that reflects graphically where the cluster is positioned in feature space, we have used the average weight from each input pixel to each CN group. If an input image comprises $\times$ by *Y* pixels, where each input pixel is represented by a group of $K_I$ neurons, the blackness $Z_{xy}$ (value 0–255) of the point (*x*, *y*) in the pseudo-digit representation for the corresponding CN group *C* is given by

11 $$\begin{aligned} Z_{xy}= \left( \frac{\sum _{i=1}^{K_I} \sum _{j=1}^{K_C} g_{xyij}}{K_I K_C}\right) \frac{Z_{\text {max}}}{g_{\text {max}}} \end{aligned}$$

where $g_{xyij}$ is the conductance of the synapse connecting the *i*th of $K_\mathrm{I}$ neurons representing input pixel (*x*, *y*) with the *j*th of $K_\mathrm{C}$ neurons representing CN group *C*. $g_{\text {max}}$ is the maximum conductance, and $Z_{\text {max}}= 255$ is the maximum blackness of an MNIST pixel.

The four $10\times 10$ grids in the left-hand column illustrate a representative evolution of the synaptic weights of the CNs across the course of training at four time points. A representative video of the process is provided in the Supplementary Information. It is evident how the initial, high random weights are pared away for low or non-firing white pixels in the input, where post-spikes are not directly preceded by pre-spikes. High firing (black) pixels in the input lead to potentiated weights. It is evident that learning is not restricted to a subset of CN groups but spreads around the full set as responding CNs are net weakened compared to as yet unused peers. It is also evident how prototypes representing variations of all 10 digits appear in the grid. However, it also clear that the distribution is not completely uniform, and it appears that sparser patterns with less overlap with other digits (e.g., the digit “1”) are somewhat favored over more populated patterns that are more likely to overlap with other digits (e.g., “0”). This is likely a consequence of not implementing any matching on white “off” pixels (see Sect. 5).

By transforming weights back into feature space, we can view clustering performance in feature space using a PCA projection and use a numerical metric to compare to other clustering methods. The right column of Fig. 3 shows, at each snapshot stage, the learned cluster points (black crosses) in a 2D PCA projection of feature space overlaying the set of data points presented up to that stage (colored digits). Note that input pixels that are not used in any input pattern retain their high initial weightings, potentially leading to a misleading weight-based rendering in feature space. We, therefore, masked out (neglected) the least active 10% of pixels across the training set. The figure shows clearly how points leave the initial closely grouped configuration and move out across feature space creating an unsupervised, self-organizing map of it.

**Fig. 4:**
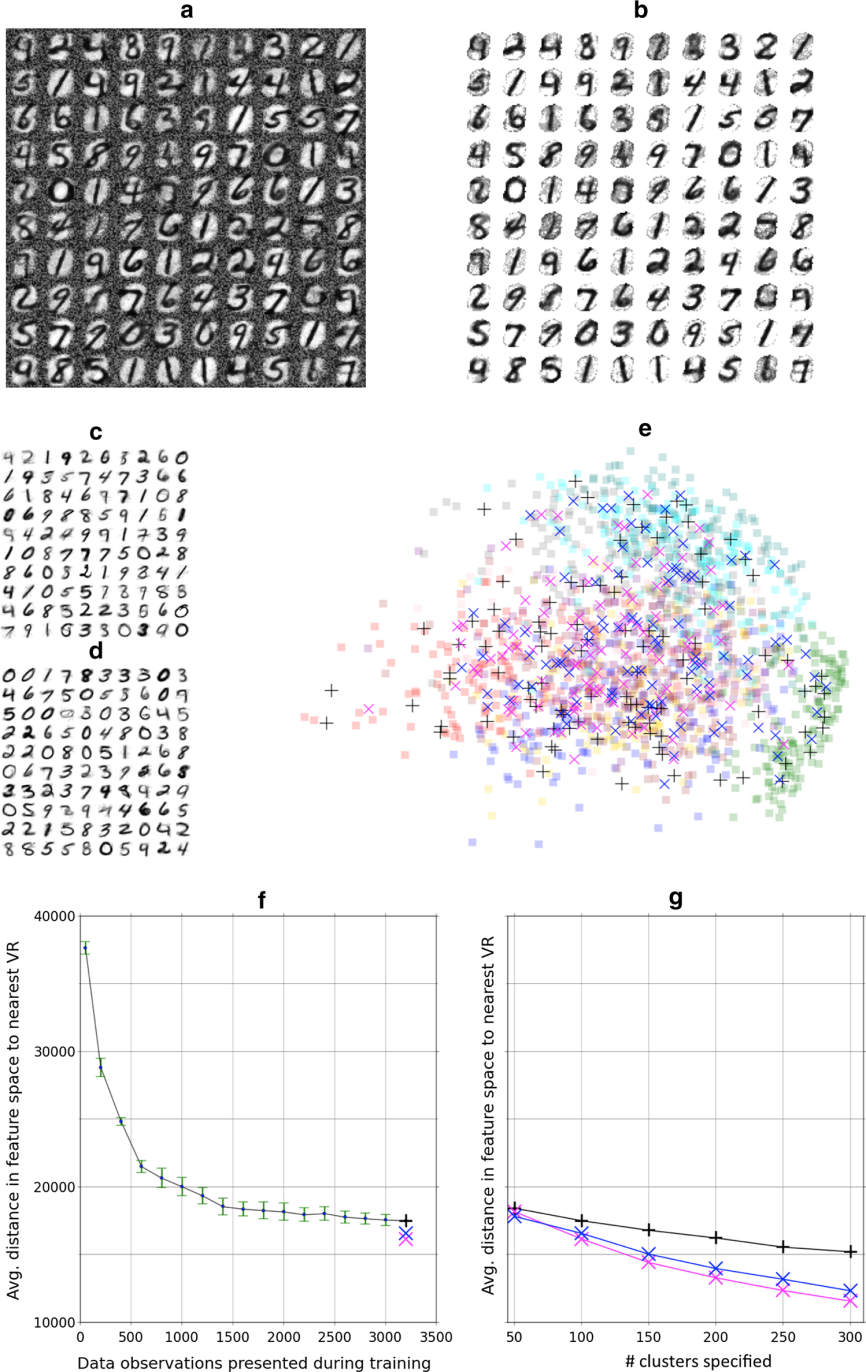
Visualization of completed VR mapping following exposure to 1600 training digits. **a** The averaged weight of plastic synapses from each rate-coded input pixel to each of 100 neuron subpopulations after a second exposure to the training set. **b** As 4A but including the masking out of the least active 10% of pixels across the 1600 training digits. This allows the learned VR points to be plotted meaningfully against the dataset. **c** Visualization of the equivalent 100 points in feature space mapped out using the standard “neural gas” algorithm against the 1600 training digits. **d** As c, but using the *k*-means clustering algorithm to place 100 cluster points. **e** The 3 sets of learned points (black crosses = model, magenta Xs = neural gas, blue Xs = *k*-means) in a 2D PCA projection of feature space overlaying the set of 1600 training data points (squares color-coded by class). **f** Quantitative comparison of effective cluster point (VR) placements across the course of training. The plot shows the average Manhattan distance (in feature space) from observations to the closest of the set of 100 VR points during the self-organization process. The final average distance (black +) achieved is comparable to that achieved by neural gas (magenta X) and *k*-means (blue X) trained for 100 clusters on the same dataset. Error bars show the standard deviation across 5 runs. **g** As f but comparing the final converged result for a range of VR numbers (numbers of cluster points) from 50 to 300

Figure 4a illustrates the final state of self-organization following two passes of the training dataset, while Fig. 4b shows the same image with masking in place, as used for the PCA plot. Compared to the earlier snapshots, the final result has qualitatively reached a distribution closer to the set of 100 cluster points in feature space generated by the neural gas algorithm (Fig. 4c) and by *k*-means clustering (Fig. 4d) against the same training set. Figure 4e plots all three sets of cluster points against the combined full training set using the 2 first components of a PCA. The figure qualitatively shows how the self-organized model has distributed its own cluster points across the whole of the feature space with representation in the region of each digit. To establish a more quantitative measure of clustering quality, we undertook two further checks. Firstly, the raw clustering ability was compared using a simplification of the model description length (Grünwald 2000; Rissanen 1978) metric; namely, we ascertained the average distance (in feature space) from each data point to the nearest cluster point:

12 $$\begin{aligned}&\overline{d}_{cluster} = \frac{1}{N_{\text {data}}} \sum _{k=1}^{N_{\text {data}}} d(\mathbf x _k, \mathbf p _{j(k)}) \end{aligned}$$

13 $$\begin{aligned}&j(k) = \underset{i}{{\text {arg min}}} (d(\mathbf x _k, \mathbf p _{i}) \end{aligned}$$

where *d* is again the Manhattan distance as in (10) and we use the pseudo-MNIST digit representation with blanked out 10% least active pixels for the cluster centers $\mathbf p _i$ of our network model. This metric reduces in both cases, trivially, when there are more cluster points but also later in training, when they become increasingly better placed. Figure 4f shows how the metric reduces over the course of self-organization in our model as the 100 cluster points are mapped out. The final value can then be compared with the same metric applied to the results of neural gas and *k*-means clustering on the same set of inputs. Figure 4g shows the metric for the three different clustering methods as the number of cluster points is varied. Performance of the spiking model can be reasonably summarized as comparable yet lower. It is, however, difficult to establish to what degree this is a fair comparison given the imprecision of the masking approach and the fact that, for the model, it is neuronal spiking that ultimately determines the response, not an implied pseudo-digit rendering based on average weight values. We therefore moved to a second quantitative test where we judge the clustering quality purely in terms of its effectiveness in the context of a neuromorphic classifier.

**Fig. 5:**
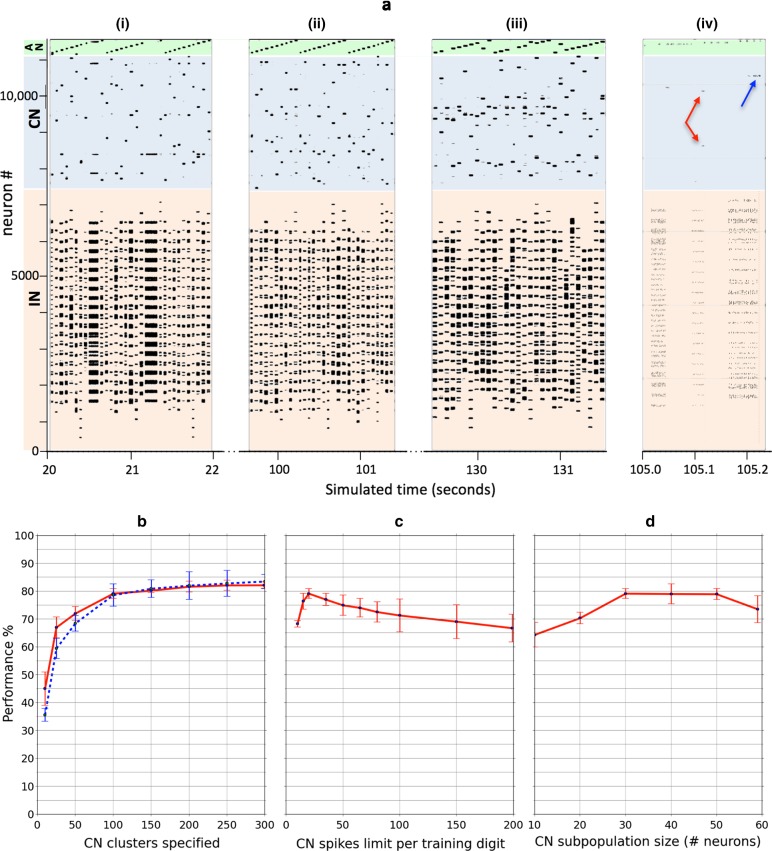
**a** Representative examples of spike raster plots comparing spiking activity in the three model layers for 30 data presentations at three stages; (i) early training, (ii) late training and (iii) testing. Training and test datasets were drawn randomly from the corresponding MNIST datasets but presented stratified in class order. A perfect “stepped” pattern in the output layer thus represents correct classification. The fourth raster plot (iv) shows a 250 ms representative detail from late training covering 3 input presentations. The red and blue superimposed arrows highlight the CN cluster responses to the second and third digit presentations included in the plot. **b** Comparing classifier performance for the previous model (Diamond et al. 2016a) (offline neural gas = blue dashed trace) and new model (online STDP self-organization = red trace) as the specified number of cluster points (aka virtual receptors) is varied. Ten thousand digits (training 1600 $\times$ 5, test 400 $\times$ 5) were drawn randomly from the corresponding MNIST datasets. For each of 5 runs, digits were presented stratified in class order. Performance is plotted as the average percentage correctly classified according to maximum output spiking activity across the 5 runs. Error bars indicate the standard deviation. The CN spike limit imposed on training was set at 20 spikes maximum, and CN cluster size was set at 30 neurons. **c** Test regime as in b, but investigating the performance impact of the CN spike limit imposed on training digit presentation in the new model. One hundred cluster points were specified, and CN cluster size was set at 30 neurons. **d** Test regime as in b, but investigating the performance impact of the CN cluster size employed in the new model. One hundred cluster points were specified, and the CN spike limit imposed during training was set at 20 spikes maximum

### Testing clustering quality with the classifier model

We combined the self-organized clustering stage with our linear classifier (Schmuker et al. 2014; Diamond et al. 2016a) (see Fig. 2d). Figure 5a shows representative spike raster plots of the model during the presentation of 30 digits one each from (i) early training, (ii) late training and (iii) testing. The fourth (iv) raster plot shows a 250 ms representative detail from late training.

The lower region of the plots, colored brown and corresponding to the IN layer, illustrates the rate-coded representation of the training digits. The plot confirms how sparsely the MNIST pixels are employed at the top and bottom of the digits (recall that MNIST digits are encoded as horizontal rows of pixels, moving from top left to bottom right). More interestingly, we clearly see the effect of the CN spike count limit as a trigger to move to the next digit. In the early stage of training [Fig. 5a(i)], where more novelty is present in the digits, we see numerous occasions where the presentation is extended in time (horizontal black streaks) until the spike count builds up to the limit for an output spike. As a result, it takes a longer period of time to traverse the 40 presented digits. In late training [Fig. 5a(ii)], these extended presentations are shorter and much less frequent, as the classifier recognizes and responds to most variations. For the most part, the presentations are rapidly traversed, with only minor modifications as a result. The testing stage [Fig. 5a(iii)] shows the use of a fixed time presentation with each digit for the same amount of time. The detail plot [Fig. 5a(iv)] shows presentations of three different durations during late training.

The center region of the plots, colored light blue and corresponding to the CN layer, shows the expected sparse response, where lateral inhibition between clusters is blocking spiking from all but the most responsive cluster(s). The detail plot [Fig. 5a(iv)] in particular provides some interesting detail. Here we see that, for the second of the inputs (MNIST training digits), the presentation was relatively short-lived. We surmise that the response was elicited by an input similar to one previously presented for which some learning (weight plasticity) has already taken place and the CN spike limit was therefore quickly reached. In this particular example, we see that a CN response was obtained in two clusters (red arrows), which compete to undertake further learning and adjustment from the new input. These two clusters apparently have both learned representations of the same digit, which was confirmed by inspection of the weight learning visualization. By contrast, we see that the third of the inputs illustrated in Fig. 5a(iv) is sustained for much longer until plasticity has acted in the single closest CN cluster and has begun to evoke an increasing response (blue arrow), which prevents activation of other clusters through lateral inhibition. The presentation ends when the CN response finally reaches the spike limit. This input must have been relatively novel to the network at that stage.

The upper region of the plots, colored light green and corresponding to the AN output layer, should ideally respond to the stratified class presentation with perfect “saw-tooth” patterns as inputs of each class are presented in turn. We see in the training stage (i–ii) that the supervised teaching signal is reliably triggering firing in the correct output subpopulation. However, in the testing stage (iii), when the teaching signal is off, there are examples where classification is incorrect, and the spiking switches to an alternative dominating cluster.

Figure 5b compares the classification results across a range of clustering resolutions for the neuromorphic model directly against those for the previous (offline neural gas-based) model. It is evident that performance of the new model consistently matches that of the previous model at higher resolutions and exceeds it at the poorest resolutions, for example, when organizing into just 10 cluster points, one for each class. This performance comparison is interesting given that the previous model has at least two distinct advantages. Firstly, using proximity (by offline Manhattan distance calculation) in feature space to generate the input rate codes means that data about which inputs are low or off are incorporated into the information used for classification. For example, for MNIST digits, the presence of white in the image center makes identification of a “zero” easier. The new classifier does not currently have this information, and only active inputs play a role in generating an output signal. We will return to this point in Discussion. Secondly, the proximity function curve (in effect, mapping distance to a rate code) is fully specifiable offline and can be precisely optimized for best performance. This function is only implicit in the new model, resulting from the spiking behavior triggered by the lateral inhibition settings of the model.

Figure 5c and d illustrate how the performance varies with two important parameters, the CN spike count limit set during training (5c) and the size of CN groups. The results indicate that performance drops as more spikes are allowed to occur during a presentation. This suggests that for smaller spike count limits overlearning is reduced and information from a wider dataset can be assimilated before the clusters become “burned in.” It is interesting that this advantage can be retained by simply limiting the amount of spiking in combination with a high learning rate and strong initial weights, rather than using longer exposures with a very low learning rate and weak initial weights. The results therefore suggest that, with the right configuration, learning can be accelerated, covering a wide dataset with short exposures while maintaining performance. We note that the performance is consistently higher when the spike limit is reduced, right down to just 20 CN spikes allowed, before falling steeply. This is likely where there are insufficient spikes across the CN layer to allow the important lateral inhibition to function effectively.

The results (Fig. 5d) also show that the group size of CNs needs to be set to at least 30 neurons to retain effective WTA inhibition between groups. We note that, beyond 50 neurons, performance also appears to dip, perhaps due to excess net inhibition between clusters.

## Discussion and conclusion

The model performed reliably as a neurmorphic clustering “algorithm” with low variance in clustering across multiple trials and is configurable in important properties such as resolution and learning rate. These quantities are very relevant for the versatility of the model, e.g., with respect to different levels of hardware capacity, numbers of features (data dimensionality) and sizes of available training datasets.

From the results with smaller subsets with less specified clusters, it is clear that this design does not necessarily require very extensive training data to function and there is the potential to set it up for fast learning, or to rapidly assimilate smaller datasets, by specifying fewer clusters. Furthermore, when compared to the “one large neuron cloud” approaches (Nowotny et al. 2005; Nessler et al. 2013), the mechanism by which it develops and adjusts clusters is relatively transparent and repeatable which means improvements can potentially be designed and added.

When combined with the neuromorphic linear classifier introduced earlier (Diamond et al. 2016a, b), the neuromorphic clustering presented here led to the same performance as achieved earlier, when using a neural gas algorithm on a classical computer for preprocessing. This suggests that the classifier performance is not affected by the subtle differences in clustering observed between the two methods.

At first glance, subpopulation sizes of 10–40 neurons for each functional unit in the model might appear excessive, in particular when datasets are very high-dimensional and/or have a large number of clusters and hence need a large number of CN groups. It would appear that models may quickly become overly large and difficult to implement and run on neuromorphic systems. However, massive parallelism of large models at low cost is at the core of modern neuromorphic computing (Merolla et al. 2014; Khan et al. 2008; Schemmel et al. 2010), such that model size and neuron and synapse numbers are only a secondary concern. The main concern is to find functional models that perform well on existing neuromorphic hardware, which has been achieved.

During training of the unsupervised clustering stage, we presented each input until a specified number of spikes was generated in the CN layer (see Sect. 4). Our results suggest that prioritizing novel inputs, which lead to less spikes initially, in this way is productive for generating a usable cluster map in a timely manner. However, this approach requires that spikes must be fed back constantly from the neuromorphic device to the host, which then can count the spikes and control when the input to the network is updated. While there is sufficient bandwidth in the tested devices to support the necessary communication without noticeably slowing down the model, this diverges from the neuromorphic ideal where the model should take on the entire task, thus drawing low power and having low dependency on connection bandwidth. A possible means to stay on-device was suggested by Deiseroth et al. (2016), where input spiking is self-inhibited, by what is, in effect, a negative feedback loop. Although this was constructed to implement a spike timing code, this principle could nevertheless be a solution that could be adapted to automatically throttle back spiking after a pre-specified number of spikes has occurred; even though the lack of feedback to the host would probably mean that the possibility to move through the data at slower or faster pace depending on the degree of novelty would likely be lost.

We presented the input to the network as Poisson processes. Spiking in biological neurons is not Poissonian but more regular (Nawrot et al. 2008; Mochizuki et al. 2016; Riehle et al. 2018). Earlier work on the neuromorphic classifier used for the final classification in this paper (Schmuker et al. 2014) found that switching to more regular gamma processes instead of Poisson input improved the classification performance. Investigating the role of (ir)regularity of input spike trains for the performance of the combined system investigated here is an interesting direction for future work.

In the model description, we noted that all plasticity remains active throughout both training and testing. We did not test scenarios where plasticity is modulated, e.g., removed sequentially as layers are trained, or removed wholly during the test stage. Whether this approach is “correct” or “best” may depend on the biological realism required although the model was certainly functional in this configuration with little to suggest that performance was being degraded. One exception is that initial investigations showed that markedly improved results are obtained if self-organization in the input layer is allowed to reach a certain level of completion before associated outputs are “burned in.” To exploit this, CN-AN weights were reset following the first epoch of training and supervised learning in the output synapses then began afresh in parallel to the continuing refinements in the first layer during the second epoch. Overall, we would like to suggest that our implementation reflects a relatively natural situation where learning is always active, in particular whenever novel information is presented.

The interest in neuromorphic hardware is driven by a desire for faster and less energy-intensive computing. We have in this paper demonstrated a neuromorphic algorithm that is able to perform unsupervised clustering on a similar level as a neural gas or *k*-means standard machine learning algorithm but entirely executed as a spiking neural network on neuromorphic hardware systems. The results of clustering are comparable to the standard methods and can be combined with a simple neuromorphic linear classifier presented earlier (Diamond et al. 2016a, b) to perform a standard benchmark classification task. This demonstrates that algorithms of general use can be implemented on neuromorphic hardware systems. Readers interested in the speed gains and power savings that can be achieved on these platforms are referred to recent work that focuses on this particular aspect (Diamond et al. 2016a; Knight and Nowotny 2018).

## Electronic supplementary material

Below is the link to the electronic supplementary material.

Supplementary material 1 (mov 22593 KB)
